# Using text analysis software to identify determinants of inappropriate clinical question reporting and diagnostic procedure referrals in Reggio Emilia, Italy

**DOI:** 10.1186/s12913-021-06093-0

**Published:** 2021-01-29

**Authors:** Francesco Venturelli, Marta Ottone, Fabio Pignatti, Eletta Bellocchio, Mirco Pinotti, Giulia Besutti, Olivera Djuric, Paolo Giorgi Rossi

**Affiliations:** 1 Epidemiology Unit, Azienda USL-IRCCS di Reggio Emilia, via Amendola 2, 42122 Reggio Emilia, Italy; 2 Clinical and Experimental Medicine PhD program, University of Modena and Reggio Emilia, Modena, Italy; 3 Department of Primary Care, Azienda USL-IRCCS di Reggio Emilia, Reggio Emilia, Italy; 4 Radiology Unit, Azienda USL-IRCCS di Reggio Emilia, Reggio Emilia, Italy

## Abstract

**Background:**

Inappropriate prescribing of diagnostic procedures leads to overdiagnosis, overtreatment and resource waste in healthcare systems. Effective strategies to measure and to overcome inappropriateness are essential to increasing the value and sustainability of care.

We aimed to describe the determinants of inappropriate reporting of the clinical question and of inappropriate imaging and endoscopy referrals through an analysis of general practitioners’ (GP) referral forms in the province of Reggio Emilia, Italy.

**Methods:**

A clinical audit was conducted on routinely collected referral forms of all GPs of Reggio Emilia province. All prescriptions for gastroscopy, colonoscopy, neurological and musculoskeletal computerised tomography (CT) and magnetic resonance imaging (MRI) from 2012 to 2017 were included. The appropriateness of referral forms was assessed using Clinika VAP software, which combines semantic analysis of clinical questions and available metadata. Local protocols agreed on by all physicians defined criteria of appropriateness. Two multilevel logistic models were used to identify multiple predictors of inappropriateness of referral forms and to analyse variability among GPs, primary care subdistricts and healthcare districts.

**Results:**

Overall, 37% of referral forms were classified as inappropriate, gastroscopy and CT showed higher proportions of inappropriate referrals compared to colonoscopy and MRI. Inappropriateness increased with patient age for CT and MRI; for gastroscopy, it was lower for patients aged 65–84 compared to those younger, and for colonoscopy, it was higher for older patients. Fee exemptions were associated with inappropriateness in MRI referral forms. The effect of GPs’ practice organization was consistent across all tests, showing higher inappropriateness for primary care medical networks than in primary care medical groups. Male GPs were associated with inappropriateness in endoscopy, and older GPs were associated with inappropriateness in musculoskeletal CT. While there was moderate variability in the inappropriate prescribing among GPs, there was not among the healthcare districts or primary care subdistricts.

**Conclusions:**

Routinely collected data and IT tools can be useful to identify and monitor diagnostic procedures at high risk of inappropriate prescribing. Assessing determinants of inappropriate referral makes it possible to tailor educational and organizational interventions to those who need them.

**Supplementary Information:**

The online version contains supplementary material available at 10.1186/s12913-021-06093-0.

## Background

The burden of inappropriate use of diagnostic procedures can be seen worldwide; its impact on patients’ health and quality of life can include overdiagnosis, overexposure to radiation, complication due to invasive procedures and overtreatment [1–3]. Moreover, its impact on sustainability is an additional issue, especially in universal health care systems, where it leads to resource waste, increase in costs and longer waiting lists [4].

Thus, tackling inappropriateness is worthwhile. To do so requires identifying its drivers; strategies to overcome barriers to appropriate prescribing would thus increase the value of care [3]. In 2017, experts from 19 countries who are members of the European Society of Radiology defined a list of critical issues and needs to increase appropriateness in the use of diagnostic imaging procedures [5], including the need for evidence-based imaging referral guidelines [5–7], the need to justify diagnostic procedures, particularly invasive procedures using ionizing radiation or contrast medium, and the need for general practitioners and specialists to work jointly [5, 8]. Another issue is that of defensive medicine, which can lead to an increase in diagnostic procedure prescriptions, although this appears to only partially explain the whole phenomenon [9]. Inappropriate prescription of gastrointestinal endoscopic procedures is also considered critical primarily for two reasons: they are invasive procedures, which cause considerable discomfort for the patient, and they have an intrinsic, although low, risk of serious complications, particularly colonoscopy [10]. Endoscopy is the main bottleneck in colorectal screening programmes in most countries; it surely is in Italy [11, 12], having become the main barrier to scaling up one of the most effective and cost-effective prevention interventions [13]. Thus, inappropriate referrals compete for allocation of resources to other, more useful and often more urgent procedures, thereby reducing the allocative efficiency of the system [14].

Although the drivers of and barriers to appropriate use of diagnostic resources are similar globally, actions should be taken at both the national and the local level [5].

In the province of Reggio Emilia (Emilia-Romagna region, northeastern Italy), a multi-component intervention has been in place since 2008 to improve the clinical and organizational appropriateness of diagnostic procedures. A multidisciplinary working group made up of all the Reggio Emilia Local Health Authority healthcare professionals and stakeholders defined a list of criteria for prescribing neurological and musculoskeletal magnetic resonance imaging (MRI) and computed tomography (CT) and for gastroscopy and colonoscopy.

This information was used by a software to define and assess specific types of misreporting and clinical inappropriateness of all referral forms for the diagnostic procedures listed above, routinely entered in an electronic database.

### Aims

The aim of this study was to describe the determinants of incomplete or meaningless reporting of the clinical question, of inappropriate imaging and endoscopy referrals and of an inappropriate level of urgency indicated in the referral through an analysis of general practitioners’ (GP) referral forms in the province of Reggio Emilia, Italy.

## Methods

### Study design

The analyses here reported are part of a comprehensive clinical audit cycle in a quality improvement system. A trend analysis of the main indicators is reported. Cross-sectional analysis of the association between inappropriateness of the prescription and patients’, GPs’ and healthcare organization’s characteristics for the period 2012–2017 was conducted on routinely collected data.

### Setting and population

This study was implemented within the Local Health Authority of Reggio Emilia, which provides healthcare services for the whole population of the province of Reggio Emilia (approximatively 530,000 inhabitants in 2019). The province is divided into 6 healthcare districts, with 23 primary care subdistricts (PCSDs) in which about 308 general practitioners (GP) operate. In each PCSD, the GPs are organised in practices sharing the same clinic and infrastructure and in which the beneficiaries are assisted by any one of the GPs in the group (primary care medical group), or where beneficiaries are assisted by their own GP, who shares some IT infrastructures with other GPs (primary care medical network) (See Additional file 1: Appendix Table 1 for details).

The Italian National Health Service is based on principles of universalism and comprehensiveness, and the Ministry of Health has the exclusive mandate to set the so-called “essential levels of care” (LEA), the list of healthcare services which must be guaranteed to all citizens and resident foreigners. The Italian Regional Healthcare Services allocate and administer the public funding for and establish the organization of providing the LEA. Only the healthcare services indicated in the LEA are available for free. In this context, GPs have the task of filtering access of their patients to specialist outpatient services, per the gatekeeping model. GPs can request various health services through a computerized referral form which should include a diagnostic question and relevant information on the clinical condition of the patient.

### Data sources

In Emilia-Romagna Region, all the computerized referral forms of outpatient care are routinely recorded in a specific database; the information collected includes the patient’s personal information, the name of the referring physician, the date of prescription, the clinical question or health condition requiring the procedure and the procedure/test requested.

In this study we analysed all the general practitioners’ referral forms in the period 2012–2017 for colonoscopy, gastroscopy, neuro CT, musculoskeletal CT, neuro MRI and musculoskeletal MRI performed by public and private providers operating for the National Health Service in the province of Reggio Emilia. Approximately 323,000 referral forms were written by GPs of the province, accounting for 85% of all referral forms in the study period.

### Intervention

In the province of Reggio Emilia, the clinical question on referral forms for diagnostic procedures has been mandatory since 2005. Moreover, in 2008, a multidisciplinary working group including all healthcare professionals and stakeholders defined a list of criteria for prescribing neurological and musculoskeletal magnetic resonance imaging (MRI) and computed tomography (CT) and for gastroscopy and colonoscopy on the basis of national and international guidelines. The criteria also defined the level of urgency (i.e. urgent (U), deferred urgency (B), to be scheduled within 60 days (D) or planned follow-up (P)) appropriate for each pair of clinical question/ requested test.

The multidisciplinary working group implemented an educational and retraining programme for GPs and specialists.

Software for the analysis of clinical questions was developed and implemented to assess the appropriateness of referral forms for colonoscopy, gastroscopy, neuro CT, musculoskeletal CT, neuro MRI and musculoskeletal MRI. This assessment was based on criteria included in the developed provincial protocols. The software uses features designed to process “unstructured clinical information” to recognize and make available the embedded knowledge items (i.e. “semantic analysis engine optimized for the ontology and thesaurus of a clinical field, with over 2 million concepts constantly updated”).

The software used for the assessment is called Clinika / VAP, produced by IG Consulting (Maps Group). It can be used for either an ex-ante assessment (i.e. to support the doctor in filling out the referral form) or an ex-post assessment (i.e. to perform periodic systematic assessments on prescribing behaviour).

The text of the clinical questions undergoes a semantic analysis to recognize and organize the clinical concepts indicated by the prescriber. All types of clinical concepts concerning “conditions/diseases” are classified and linked with the appropriate domain language. Subsequently, the prescribing rules are interpolated in the diagnostic procedure required in order to verify that the condition/disease identified in the clinical question is one of those admitted by the protocol and to obtain the priority admitted by the protocol for this type of combination (i.e. diagnostic procedure – condition/disease). The priority requested on the referral form and the priority foreseen by the protocol are then compared.

Benchmarking reports are periodically produced and sent to GPs and coordinators of primary care subdistricts to motivate GPs to improve the appropriateness of their diagnostic procedure referral practices.

The appropriateness of diagnostic procedure referral has also been included among the objectives agreed on by the LHA and the primary care subdistricts for granting GPs incentives.

### Endpoints

**Fig. 1:**
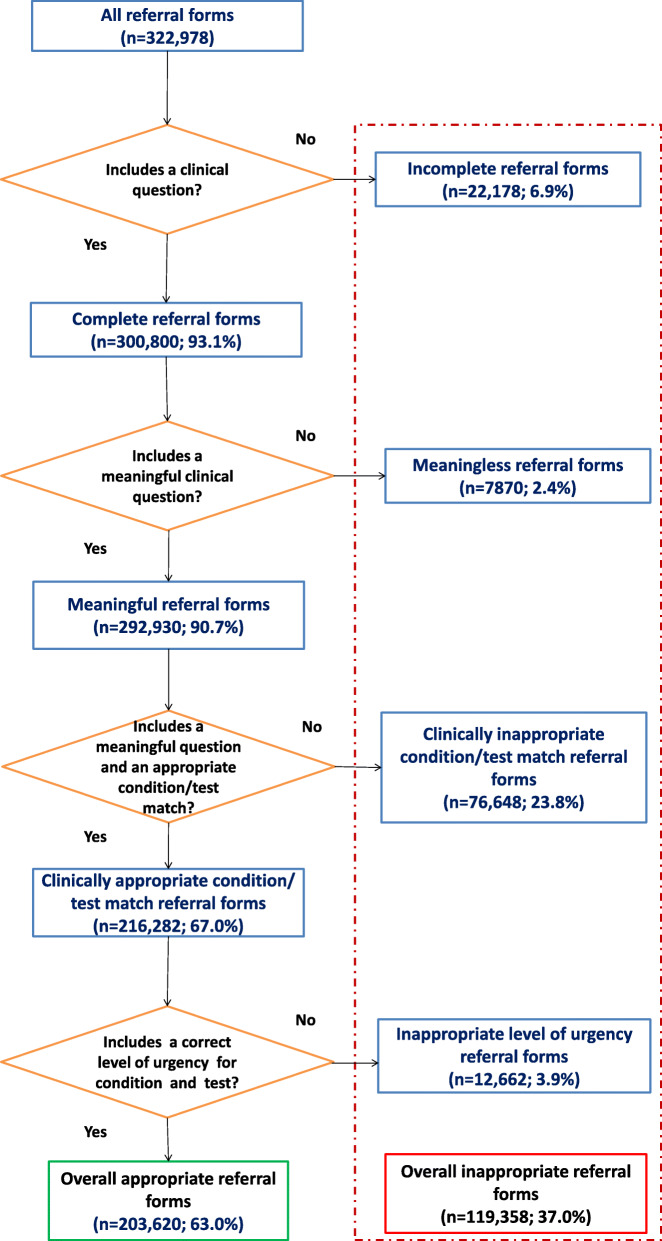
Flowchart of ex-post assessment of overall inappropriateness of referral forms using the Clinika/VAP software. Data shown are related to all referral forms for all six of the included diagnostic procedures. The percentage values refer to the total number of referral forms (i.e. *n* = 322,978) prescribed by general practitioners in the province of Reggio Emilia, Italy, between 2012 and 2017

This process assigns each referral form, according to characteristics assessed by the software, to one of the following categories (Fig. 1):

- Inappropriate:
  - clinical question missing (i.e. incomplete);
  - clinical question present, but with no clinical meaning (i.e. meaningless);
  - meaningful clinical question not matching any criterion included in the protocol for the diagnostic procedure prescribed (i.e. clinically inappropriate condition/test match);
  - clinically appropriate condition/test match but priority level different from that foreseen for the health problem (i.e. inappropriate level of urgency)
- Appropriate:
  - Clinical question and priority level consistent with the health problem, as indicated by the protocols for the diagnostic procedure prescribed.

The primary endpoint was overall appropriateness, including all missing, meaningless reporting, clinically inappropriate condition/test match and inappropriate level of urgency. The secondary endpoint was to also evaluate only the clinically inappropriate test/condition match, excluding incomplete and meaningless referrals from the analysis. In this analysis, referrals with an inappropriate level of urgency were included among inappropriate referrals because, while this type of inappropriateness is independent of clinical reasons, it does lead to allocative inefficiency and inappropriate use of resources.

An appropriateness evaluation performed by Clinika software and an expert radiologist or gastroenterologist on a sample of 100 records for endoscopy procedures showed an agreement of 78.8% (K = 0.64) for colonoscopy and 85.9% (K = 0.66) for gastroscopy.

### Statistical analysis

Descriptive analyses of referral forms were performed for each type of diagnostic procedure prescribed and according to predefined determinants of inappropriateness.

Time trends were calculated for each prescribed diagnostic procedure between 2012 and 2017.

The determinants of overall appropriateness were calendar year, whether a health fee waiver (exemption) was applied (providing diagnostic procedure for free), age and sex of patient and of physician and GP’s practice organization (primary care medical group or primary care medical network).

Random intercept multilevel models that included GPs, primary care subdistricts and healthcare districts as random effects were made [15]. We report the fixed effect of the models: the adjusted odds ratios (ORs) from the multilevel models that included patient-level factors and GP-level factors to identify determinants of the overall inappropriateness of referral forms. Furthermore, we used a multilevel model adjusted for 1st level variables to analyse the variability in inappropriateness of referral forms between GPs (level 2), between primary care subdistricts (level 3) and between healthcare districts (level 4). We summarized the residual variability and interclass correlation coefficients (ICC), which represents the proportion of the total variance in inappropriateness directly attributable to each level after taking into account the division random effects and fixed effects for all variables.

The variability in appropriateness was then assessed at three levels: GP, primary care subdistrict and healthcare district. The multilevel logistic model was used to assess the influence of individual predictors on inappropriateness of referral forms. Let [*P*[*Y*_*ijkl*_ = 1]] be the probability that the lth referral form ordered by the kth general practitioner in the j^th^ primary care subdistrict in the i^th^ healthcare district was inappropriate. The model is written out as follows:

$$ logit\left[P\left[{Y}_{ijkl}=1\right]\right]=\mathit{\log}\left[\frac{P\left[{Y}_{ijkl}=1\right]}{1-P\left[{Y}_{ijkl}=1\right]}\right]=\left({\beta}_{0i}+{\beta}_{0j}+{\beta}_{0k}\right)+\theta {X}_{ijkl} $$

where *β*_0*i*_ is the healthcare district-specific random intercept, *β*_0*j*_ the primary care subdistrict-specific random intercept, *β*_0*k*_ the GP-specific random intercept, *X*_*ijkl*_ the vector of individual-level covariates and *θ* the vector of individual-level coefficients.

We used STATA 13.0SE (Stata Corporation, Texas, TX) software package for the main analysis.

### Ethics

This is a clinical audit on routinely collected administrative data. According to the Italian law, clinical audits do not require the approval of an ethics committee. Data are available upon reasonable request by writing to info.epi@ausl.re.it.

## Results

### Descriptive analysis

Overall, 322,978 referral forms were included in the analysis, of which 203,620 (63.0%) proved to be appropriate according to our assessment criteria. Inappropriateness was mainly driven by clinical inappropriateness (i.e. mismatch between health problem, diagnostic procedure prescribed and level of urgency), accounting for 27.7% (*n* = 89,310/322,978) of all prescribed referral forms, while inappropriate reporting of clinical question (i.e. presence of a clinical question that defines a clear health problem included among the prescribing criteria) accounted for 9.3% (*n* = 30,048/322,978) of overall inappropriateness (Fig. 1).

**Fig. 2:**
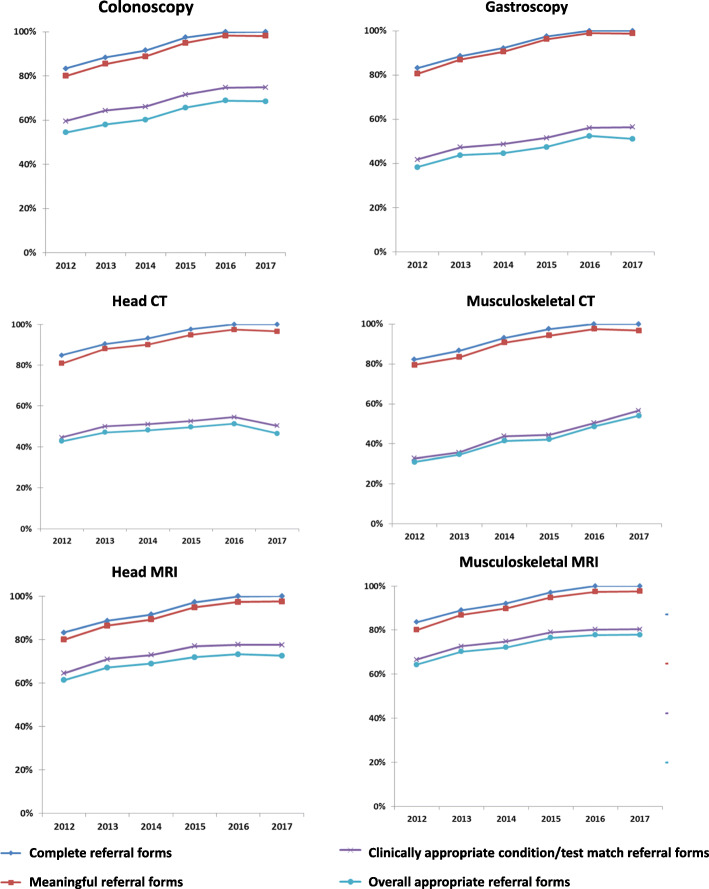
Trends in proportion of completeness, meaningful, clinically appropriate condition/test match and overall appropriateness of referral forms prescribed by general practitioners from 2012 to 2017 in the province of Reggio Emilia, Italy, using the Clinika /AVP software by diagnostic procedure

The same pattern was found among referral forms for each diagnostic procedure, with greater clinical inappropriateness for endoscopy and CT than for MRI. Assessing referral forms by diagnostic procedure and year of prescription, we observed an overall decrease in inappropriateness, from 44.2% in 2012 to 32.9% in 2017. In particular, an increasing trend (from 2012 to 2016) for all appropriateness endpoints occurred for all diagnostic procedures, with a substantial plateau between 2016 and 2017. Only neuro CT showed a slight reduction in appropriateness in the last year of the observation period, while musculoskeletal CT appropriateness rose during over same period (Fig. 2).

The number of referral forms and the overall proportion of inappropriateness varied according to patient, prescription and prescribing physician’s characteristics (Table 1). The lowest proportion of appropriateness was generally found among referral forms provided to older people (i.e. over age 84) and to female patients, with the exception of gastroscopy prescriptions, for which appropriateness was lower in males and in younger patients. Further, the overall proportion of appropriateness decreased with the increasing of the level of urgency, while a similar proportion was found between referral forms including or not including fee exemptions. Regarding prescribers’ characteristics, the lowest proportion of appropriateness was found among prescriptions provided by male and by older physicians. GPs working in a medical network wrote fewer appropriate prescriptions than did GPs working in a medical group. Differences in the proportions of appropriate referral forms were found among the six health districts of the province.

**Table 1:** Descriptive data for patients’, physicians’ and prescriptions’ characteristics

	Colonoscopy		Gastroscopy		Head CT		Musculoskeletal CT		Head MRI		Musculoskeletal MRI		Overall	
	Total	Inappr.	Total	Inappr.	Total	Inappr.	Total	Inappr.	Total	Inappr.	Total	Inappr.	Total	Inappr.
**Overall**	52,583	37.3%	51,895	53.7%	24,029	52.6%	5221	58.6%	109,689	31.3%	79,561	27.5%	322,978	37,0%
**Patient’s age**														
*15–64*	31,859	37.4%	35,769	58.8%	13,068	50.0%	3543	57.1%	82,844	30.3%	64,883	27.2%	231,966	36.3%
*65–84*	19,608	36.6%	15,082	41.5%	9554	55.2%	1571	62.3%	25,740	34.2%	14,319	28.6%	85,874	37.9%
*> 84*	1116	46.7%	1044	56.1%	1407	58.8%	107	57.0%	1105	38.5%	358	41.3%	5137	50,0%
**Patient’s sex**														
*M*	24,542	35.8%	22,650	56.0%	10,856	53.6%	2415	57.3%	47,643	30.6%	41,388	27.0%	126,844	25.1%
*F*	28,038	38.6%	29,243	51.9%	13,173	51.7%	2806	59.8%	62,042	31.8%	38,168	28.0%	173,470	37.4%
**Level of urgency**														
*Urgent (U)*	205	100%	272	100%	278	84.9%	69	100%	553	100%	277	100%	1654	97.5%
*Deferred urgency (B)*	4516	96.3%	4260	94.3%	2664	66.8%	491	48.7%	6077	92.8%	2568	92.0%	20,576	89.4%
*Scheduled within 60 days (D)*	44,335	32.6%	45,155	49.8%	20,226	50.3%	4,515	59.4%	98,588	27.1%	74,369	25.2%	287,188	33.2%
*Scheduled follow up (P)*	3527	17.4%	2208	49.0%	861	52.4%	146	50.0%	4,471	29.9%	2347	20.7%	13,560	29.8%
**Exemption**														
*Providing test for free*	16,694	36.3%	12,403	44.5%	8371	54.0%	1324	57.9%	21,929	35.8%	10,731	28.2%	71,452	38.8%
*Not providing test for free*	35,889	37.8%	39,492	56.6%	15,658	51.8%	3897	58.9%	87,760	30.1%	68,830	27.4%	251,526	36.4%
**Year of referral**														
*2012*	8237	45.6%	8542	61.7%	4572	57.3%	993	69.0%	20,816	38.8%	15,842	35.6%	59,002	44.2%
*2013*	8816	42.0%	8732	56.2%	3915	52.9%	918	65.4%	18,552	32.9%	14,095	29.8%	55,028	39.2%
*2014*	8897	39.8%	8927	55.4%	4025	51.8%	913	58.5%	19,861	31.2%	14,729	27.9%	57,352	37.3%
*2015*	8751	34.4%	8387	52.6%	4138	50.3%	863	57.8%	19,072	28.2%	13,555	23.5%	54,766	33.9%
*2016*	8589	31.2%	8298	47.6%	3505	48.7%	718	51.4%	14,141	26.8%	9553	22.3%	44,804	32.6%
*2017*	9293	31.5%	9009	48.8%	3874	53.5%	816	46.0%	17,247	27.5%	11,787	22.2%	52,026	32.9%
**Physician’s sex**														
*M*	36,125	39.6%	35,209	54.7%	16,940	52.9%	3661	59.9%	73,697	32.1%	54,699	28.1%	220,331	38.0%
*F*	16,107	32.4%	16,336	51.6%	6933	51.6%	1515	55.4%	35,132	29.3%	24,282	26.1%	100,305	34.6%
**Physician’s age**														
*28–44*	3390	29.3%	3209	48.2%	1473	50.0%	317	44.8%	7003	27.4%	4528	24.8%	19,920	32.5%
*45–68*	48,842	37.9%	48,336	54.0%	22,400	52.7%	4859	59.5%	101,826	31.5%	74,453	27.6%	300,716	37.2%
GPs’ practice organization*														
*Primary care medical group*	14,822	35.5%	14,205	49.4%	6921	51.4%	1448	54.5%	31,600	27.8%	23,541	23.8%	92,537	33.5%
*Primary care medical network*	33,736	38.5%	33,654	55.8%	15,357	52.9%	3373	60.3%	69,829	32.9%	50,631	29.4%	206,580	38.6%
**District of physician**														
*Mountains*	2350	39.4%	3345	54.6%	1635	59.0%	303	57.4%	5753	36.5%	3449	29.9%	16,835	41.7%
*Eastern plains*	6313	32.2%	6346	51.2%	2566	50.4%	536	52.1%	11,676	26.8%	9018	24.2%	36,455	33.4%
*Northern plains*	6517	37.6%	7048	54.5%	3721	52.1%	785	58.0%	15,967	31.7%	12,116	27.5%	46,154	37.0%
*Western hills*	5785	40.0%	5381	54.0%	3170	51.0%	618	64.6%	11,634	32.3%	8924	29.9%	35,512	38.5%
*Provincial capital*	24,631	39.1%	22,602	55.4%	9483	53.4%	2242	61.2%	48,184	32.4%	34,069	29.0%	141,211	38.3%
*Eastern hills*	6636	32.3%	6823	48.9%	3298	50.6%	692	51.2%	15,615	27.8%	11,405	22.9%	44,469	32.5%

Data expressed as n and percentage, by diagnostic procedure and overall, for all referral forms made by general practitioners in the province of Reggio Emilia, Italy, between 2012 and 2017

### Determinants of inappropriateness

Inappropriateness increased with patient age for all imaging procedures, while a lower risk of inappropriateness of gastroscopy was found among patients aged 65–84. Moreover, compared to female patients, male patients showed a lower risk of inappropriateness for colonoscopy, musculoskeletal CT, neuro MRI and musculoskeletal MRI and a higher risk for gastroscopy and neuro CT. Referral forms with fee exemptions resulted in greater inappropriateness for musculoskeletal MRI and neuro MRI. For all diagnostic procedures, inappropriateness of prescription was strongly associated with the GP’s work structure. Regarding physician characteristics, male physicians were positively associated with inappropriate colonoscopy (OR = 1.33; 95% CI = 1.14–1.56) and gastroscopy (OR = 1.18; 95% CI = 1.02–1.36), while older physicians were positively associated with inappropriate musculoskeletal CT (OR = 2.17; 95% CI = 1.30–3.62) (Table 2).

**Table 2:** Multilevel analysis of overall inappropriateness of referral forms: (A) fixed effects; (B) random effects estimates

**(A) Risk of overall inappropriateness**	**Colonoscopy**			**Gastroscopy**			**Head CT**			**Musculoskeletal CT**			**Head MRI**			**Musculoskeletal MRI**		
	**OR**	**95%CI**		**OR**	**95% CI**		**OR**	**95% CI**		**OR**	**95% CI**		**OR**	**95% CI**		**OR**	**95% CI**	
***Year of prescription***																		
*2012*	1.00	–	–	1.00	–	–	1.00	–	–	1.00	–	–	1.00	–	–	1.00	–	–
*2013*	**0.82**	**0.76**	**0.87**	**0.78**	**0.72**	**0.83**	**0.83**	**0.75**	**0.91**	0.86	0.69	1.07	0.68	0.65	0.71	**0.71**	**0.67**	**0.75**
*2014*	**0.74**	**0.69**	**0.79**	**0.75**	**0.70**	**0.80**	**0.81**	**0.74**	**0.90**	**0.64**	**0.52**	**0.80**	0.62	0.59	0.65	**0.64**	**0.61**	**0.67**
*2015*	**0.59**	**0.55**	**0.63**	**0.66**	**0.61**	**0.70**	**0.74**	**0.67**	**0.82**	**0.58**	**0.46**	**0.72**	0.53	0.51	0.56	**0.50**	**0.48**	**0.53**
*2016*	**0.51**	**0.47**	**0.55**	**0.53**	**0.49**	**0.57**	**0.67**	**0.60**	**0.74**	**0.47**	**0.37**	**0.60**	0.49	0.46	0.51	**0.43**	**0.40**	**0.46**
*2017*	**0.50**	**0.47**	**0.54**	**0.58**	**0.54**	**0.62**	**0.81**	**0.73**	**0.90**	**0.38**	**0.30**	**0.48**	0.49	0.46	0.51	**0.43**	**0.41**	**0.46**
**Patient’s age**																		
*15–64*	1.00			1.00			1.00			1.00			1.00			1.00		
*65–84*	0.97	0.92	1.01	**0.49**	**0.47**	**0.52**	**1.26**	**1.18**	**1.36**	**1.30**	**1.10**	**1.54**	1.02	0.98	1.06	1.04	0.98	1.09
*> 84*	**1.56**	**1.36**	**1.79**	0.89	0.77	1.02	**1.48**	**1.30**	**1.69**	1.01	0.64	1.61	1.10	0.96	1.27	**1.86**	**1.46**	**2.38**
**Patient’s sex**																		
*F*	1.00			1.00			1.00			1.00			1.00			1.00		
*M*	**0.85**	**0.82**	**0.88**	**1.19**	**1.14**	**1.23**	**1.07**	**1.01**	**1.13**	**0.82**	**0.72**	**0.94**	**0.94**	**0.92**	**0.97**	**0.92**	**0.89**	**0.96**
**Fee exemption**																		
*No*	1.00			1.00			1.00			1.00			1.00			1.00		
*Yes*	1.01	0.96	1.07	0.98	0.92	1.03	1.00	0.92	1.08	0.90	0.74	1.08	**1.45**	**1.39**	**1.51**	**1.12**	**1.06**	**1.20**
**Physician’s sex**																		
*F*	1.00			1.00			1.00			1.00			1.00			1.00		
*M*	**1.33**	**1.14**	**1.56**	**1.18**	**1.02**	**1.36**	1.05	0.92	1.21	1.16	0.92	1.48	1.09	0.93	1.26	1.09	0.92	1.29
**Physician’s age**																		
*28–44*	1.00			1.00			1.00			1.00			1.00			1.00		
*45–68*	1.14	0.85	1.53	1.08	0.82	1.43	1.07	0.82	1.40	**2.17**	**1.30**	**3.62**	0.94	0.73	1.21	0.99	0.72	1.34
***GPs’ practice organization***																		
*Primary care medical group*	1.00			1.00			1.00			1.00			1.00			1.00		
*Primary care medical network*	**1.39**	**1.16**	**1.67**	**1.40**	**1.20**	**1.63**	1.15	1.00	1.32	**1.43**	**1.11**	**1.84**	**1.36**	**1.14**	**1.63**	**1.40**	**1.15**	**1.69**
**(B) Variances of random effects** ^a^	**Variance**		**ICC %**	**Variance**		**ICC %**	**Variance**		**ICC %**	**Variance**		**ICC %**	**Variance**		**ICC %**	**Variance**		**ICC %**
*Level 2 (GP)*	0.441		11.7	0.345		9.4	0.252		7.1	0.556		14.3	0.370		9.8	0.498		12.8
*Level 3 (primary care subdistrict)*	0.037		1.0	0.031		0.8	0.000		0.0	0.027		0.7	0.095		2.5	0.090		2.3
*Level 4 (healthcare district)*	0.011		0.3	0.007		0.2	0.000		0.0	0.026		0.7	0.000		0.0	0.000		0.0

Logistic regression adjusted odds ratio and 95% CI of overall inappropriateness of referral forms for each diagnostic procedure by patient, referral form and physician variables, and analysis of the variability in overall inappropriateness of referral forms between GPs, primary care subdistrict and healthcare districts. All referral forms made out by general practitioners in the province of Reggio Emilia, Italy, between 2012 and 2017 were included

*OR* odds ratio, *ICC* interclass correlation coefficient

^a^ Model adjusted for 1st level covariates: Patient’s Age, Sex, Exemption, Year of prescription

For all diagnostic procedures, variability between GPs accounted for the highest percentage of total variability in inappropriateness (from 7.1% for neuro CT to 14.3% for musculoskeletal CT), while variability between primary care subdistricts (from 0.0% for neuro CT to 2.5% for head MRI) and healthcare district (from 0.% for MRI and neuro CT to 0.7% for musculoskeletal CT) represented a smaller portion of the total variability in inappropriateness in the statistical model adjusted for patient’s age, sex, year of prescription and exemption status.

We performed a sensitivity analysis to examine the clinical appropriateness of referral forms, excluding incomplete and meaningless referral forms (See Additional file 1: Appendix Table 2–3). The associations were almost all in the same direction of that observed for overall appropriateness, even if in most cases the associations were weaker and estimates less precise. The few exceptions are a negative association for fee exemption in endoscopy (OR = 0.88; 95% CI = 0.83–0.94 for colonoscopy and OR = 0.90; 95% CI = 0.85–0.96 for gastroscopy) and an association with older patient age for colonoscopy, which disappeared.

Variability between GPs in clinical inappropriateness was slightly smaller than in the main analysis, but its stronger effect remained among the hierarchical levels.

## Discussion

More than one-third of prescribed diagnostic procedures in our study were classified as inappropriate, with gastroscopy, neuro CT and musculoskeletal CT having a higher percentage of inappropriate referrals than colonoscopy, neuro MRI and musculoskeletal MRI. Overall, inappropriateness was mostly attributed to clinical inappropriateness (i.e. inconsistency between test, clinical question and urgency reported in the forms, according to prespecified criteria) and less to the inappropriate reporting of clinical question for referral (i.e. “incomplete” or “meaningless” forms). Despite the fact that the variability between GPs was the greatest source of inappropriateness variation, promising improvement was observed over time for all procedures under study, consistent with the implementation of several measures of training, shared protocol definition and administrative control. Both the appropriateness of requesting a diagnostic test and the clinical relevance of test results to the management of a patient require reliable, effective communication between the referring physician and the specialist [3, 5, 16]; the cornerstone of that communication is the quality and structure of a report, which includes all findings and the specialist’s interpretation of the same [16, 17]. Indeed, the report is clearly influenced by the quality of the referral form itself, mainly in terms of the accuracy and completeness of the clinical information reported by the referring physician in the clinical question [17–19].

The appropriateness of a diagnostic procedure for a given individual is not always easy to determine because most recommendations, when available and if well formulated, are conditional upon many circumstances that are difficult to assess retrospectively [20]. Many attempts have been made to measure the inappropriateness of diagnostic procedures, mainly based on the assessment of “unwarranted” geographical variability in diagnostic services use, although the validity of this approach is still debated [21, 22]. Furthermore, this type of analysis can determine that a certain level of inappropriateness is present but cannot precisely identify where the problem lies.

Rates of inappropriate imaging diagnostic procedure use in the primary care setting vary considerably (from 0.2 to 99.9%) [23], even for a single procedure within the same country (i.e. from 2 to 28.5% inappropriate MRI in Canada) [24]. Given the substantial heterogeneity of the methods applied, the observed variability could possibly reflect the appreciable variation in determinants of deviation from guidelines. Bearing in mind these substantial methodological variations, overall inappropriateness of referrals in our study was similar to that reported in a Spanish cross-sectional study (31.4%) [2], but higher than that reported in Sweden (20%) [25] and substantially higher than that found in the UK (0.3%) [26] and Finland (7%) [27], which have implemented mandatory vetting of all requested radiation-related procedures.

In our study inappropriateness mostly stemmed from the mismatch of clinically meaningful questions with a recommended type of diagnostic procedure and was especially manifest for endoscopy and CT scans, while MRI was less affected by clinical inappropriateness, as already shown in other countries [28]. The narrower range of clinical indications for referral and increased GP awareness of the need for an adequate justification imposed in recent years by the economic impact of MRI prescribing [29] may explain part of the difference between MRI and the other imaging procedures considered. Apparently, the lower costs of CT and its wider availability are enough to counterbalance the higher radiation exposure, which should actually discourage unnecessary referrals.

Besides inconsistent matching between recommendations and protocols, this step in the appropriateness flow could also be influenced by the inconsistency between the clinical condition and the diagnostic test, in particular the erroneous choice between two alternative tests, for example between CT and MRI, which can be difficult for GPs to make without the guidance of a neurologist or musculoskeletal specialist. For instance, it has been reported that, despite the recommendations of clinical societies, the use of head CT for chronic headache or of spinal CT for acute non-specific low back pain without urgent symptoms is still a source of inappropriateness [26, 30].

In our study, only 2.5% of prescriptions flagged as urgent by the GPs were classified as appropriate, and with the exception of a small percentage (15%) of appropriate urgent neuro CT, inappropriateness of all other procedures under study was 100%. The reason for such a high rate of referrals automatically assigned as inappropriate is that according to the local prescription guidelines, all urgent conditions requiring endoscopy, CT or MRI are subject to urgent referral to the emergency unit for further evaluation and care, without the GP requesting any particular procedure. The exception to this is urgent requests for neuro CT in the presence of cranial trauma or signs and symptoms of transitory ischemic attack (TIA), resulting in a small percentage of appropriate neuro CT referrals. Inappropriate levels of urgency (given the test/question match, according to the agreed-on protocols) lead to allocative inefficiency and to the inappropriate use of resources.

Our study showed that the most important determinants of inappropriate prescribing were individual characteristics of the GP, while the organizational and managerial interventions had a smaller impact, even when they involved negotiating budgets for additional incentives and education, i.e. the primary care subdistrict level, or when they involved coordinating and integrating GPs’ activities through the healthcare districts. This is in line with the result that most of the variability seen is explained by the individual GPs, with only a minor part by the healthcare district or the primary care subdistrict. These finding suggests that, at least in our organizational context, managers at healthcare district level and GP coordinators at the primary care subdistrict level had limited impact on the appropriateness of diagnostic procedure prescribing, despite the considerable autonomy in budget definition and planning these two levels have. Actions individually targeting GPs, such as training and education to disseminate tools for implementing recommendations, may be the key to reduce variability at the GP level. However, our study is one of the first to assess the impact of the medical group organizational model on GPs’ performance. The consistent results across all diagnostic procedures assessed, with primary care medical groups showing a higher level of overall appropriateness and, although to a lesser degree, of clinical appropriateness, highlight the idea that new organizational models may favourably impact quality of care.

### Strengths and limitations

As referral forms filled out by GPs were automatically assessed by a dedicated software, this study has intrinsic limitations. Given that the software’s ability to interpret the text is imperfect, misclassification of the consistency of the clinical question certainly occurred in some cases. However, the software proved to have substantial agreement with a manual review by an expert clinician, with kappa values over 0.6. Furthermore, the application of a single method to all referrals permitted a uniform assessment. As in the panel audit studies, the software assessment of overall appropriateness is limited to what is reported on the referral form; no direct information of a given patient’s health condition is available. Indeed, we could only assess the consistency between the clinical question as reported in the form, the requested test and the level of urgency attributed; we had no way to assess the trueness of any of the conditions reported or whether other unreported conditions could justify the choice of test and/ or the level of urgency. Therefore, we measured the consistency between what was reported and the agreed-on criteria, meaning that there could be appropriate referral forms that appear to be inappropriate because of incomplete reporting as well as inappropriate referral forms that appear to be appropriate because of incorrect reporting of conditions. Lastly, the assessment of appropriateness was based on a list of criteria created by the local professionals and was thus potentially limited by its incompleteness. This limitation is common to the vast majority of studies aiming to measure inappropriateness of diagnostic procedure prescribing using operational definitions of guidelines recommendations. In our study, we were not able to identify and assess “clinical inappropriateness”, or malpractice more generally, when formulating the clinical question in diagnostic prescriptions. Therefore, we considered incomplete, meaningless and clinical inappropriate referral forms together because a correctly filled prescription is a prerequisite for any monitoring and quality improvement system.

The sensitivity analysis, which examined the clinical appropriateness of referral forms, excluded those prescriptions that were not correctly completed (incomplete and/ or meaningless). It is worth noting that this indicator dramatically overestimated the proportion of clinically appropriate prescriptions: prescriptions that are not correctly completed or not completed at all were much more likely to be inappropriate than those that were completed and meaningful.

All these limitations mean that interpreting the prevalence of inappropriate prescriptions as an absolute measure of the quality of outpatient diagnostic procedure prescribing is not appropriate. Further, they resulted in an incorrect comparison with other studies due to the heterogeneity of the methods applied and the constrained generalisability of each of these designs. Instead, our method makes it possible to assess trends and to which conditions and characteristics of patients and GPs may favour inappropriateness.

## Conclusions

Despite the promising increase in overall appropriateness of diagnostic procedure prescribing over the last five years in Reggio Emilia province, there is still room for improvement. Tailored interventions to increase GP compliance to guideline recommendations should be advocated. Improved compliance to protocols would reduce the number of unnecessary tests, the radiation exposure and the risk of adverse effects, thereby improving clinical practice and lowering healthcare costs. Interventions aimed at encouraging the uptake of new organizational models of care among GPs, such as primary care medical groups, could limit the effect GPs’ characteristics have on the variability of their prescribing practices.

## Supplementary Information

**Additional file 1: Appendix Table 1.** Characteristics of a primary care medical group and a medical network in in Emilia-Romagna Region, Italy. **Appendix Table 2.** Multilevel logistic regression adjusted Odds Ratio and 95% CI of inconsistence criteria or test of referral forms for each diagnostic procedure by individual and physician variables: fixed effects estimates. All referral forms made by general practitioners in the province of Reggio Emilia, Italy, between 2012 and 2017 were included. **Appendix Table 3.** Multilevel analysis of the variability in inconsistence criteria or test of referral forms between GPs, primary care subdistrict and healthcare districts: random effects estimates. All referral forms made by general practitioners in the province of Reggio Emilia, Italy, between 2012 and 2017 were included.

## Data Availability

The datasets used during the current study are available from the corresponding author on a reasonable request.
