# Supplementary material for: Using text analysis software to identify determinants of inappropriate clinical question reporting and diagnostic procedure referrals in Reggio Emilia, Italy
Source: BMC Health Serv Res. 2021 Jan 29;21:103. doi: 10.1186/s12913-021-06093-0 (PMC7847028; doi:10.1186/s12913-021-06093-0)
Supplement: Supplementary file 1 — Additional file 1: Appendix Table 1. Characteristics of a primary care medical group and a medical network in in Emilia-Romagna Region, Italy. Appendix Table 2. Multilevel logistic regression adjusted Odds Ratio and 95% CI of inconsistence criteria or test of referral forms for each diagnostic procedure by individual and physician variables: fixed effects estimates. All referral forms made by general practitioners in the province of Reggio Emilia, Italy, between 2012 and 2017 were included. Appendix Table 3. Multilevel analysis of the variability in inconsistence criteria or test of referral forms between GPs, primary care subdistrict and healthcare districts: random effects estimates. All referral forms made by general practitioners in the province of Reggio Emilia, Italy, between 2012 and 2017 were included. [file 12913_2021_6093_MOESM1_ESM.docx]

**Appendix**

Appendix Table 1 **-** characteristics of a primary care medical group and a medical network in in Emilia-Romagna Region, Italy.

| **Primary care medical network**  **(*Medicina di Rete*)** | - general practitioners’ offices are located in the same primary care subdistrict (*Nucleo di Cure Primarie*); - a software is used to manage patients’ electronic health records - the medical network uses IT to access to information related to each of that medical network’s physicians - each doctor shares and uses epidemiological and clinical data for clinical audit and benchmarking, with the aims of improving quality of care and reaching budget targets - the network includes from 3 to 10 GPs (except in special cases); |
| --- | --- |
| **Primary care medical group**  **(*Medicina di Gruppo*)** | - the GPs, each with a private office, work together at the same physical location. - the number of offices equalling at least half of the doctors who make up the group itself, with the possibility of sharing the same office at different times; - the GPs share IT, office equipment and medical devices and technology. - the GPs share secretarial services and/ or nursing staff - a compatible software is used to manage patients’ electronic health records - The medical group uses IT to access to information related to each of that medical group’s physicians. - each doctor shares and uses epidemiological and clinical data for clinical audit and benchmarking, with the aims of improving quality of care and reaching budget targets - the group includes from 3 to 8 GPs (except in special cases) |

Appendix Table 2 **-** Multilevel logistic regression adjusted Odds Ratio and 95% CI of inconsistence criteria or test of referral forms for each diagnostic procedure by individual and physician variables: fixed effects estimates. All referral forms made by general practitioners in the province of Reggio Emilia, Italy, between 2012 and 2017 were included.

| **Risk of inconsistence** | **Colonoscopy** | | | **Gastroscopy** | | | **Head CT** | | | **Musculoskeletal CT** | | | **Head MRI** | | | **Musculoskeletal MRI** | | |
| --- | --- | --- | --- | --- | --- | --- | --- | --- | --- | --- | --- | --- | --- | --- | --- | --- | --- | --- |
|  | **OR** | **95%CI** | | **OR** | **95% CI** | | **OR** | **95% CI** | | **OR** | **95% CI** | | **OR** | **95% CI** | | **OR** | **95% CI** | |
| **Patient’s age** |  |  |  |  |  |  |  |  |  |  |  |  |  |  |  |  |  |  |
| *15-64* | 1.00 |  |  | 1.00 |  |  | 1.00 |  |  | 1.00 |  |  | 1.00 |  |  | 1.00 |  |  |
| *65-84* | **0.91** | **0.86** | **0.96** | **0.41** | **0.38** | **0.43** | **1.31** | **1.21** | **1.41** | **1.26** | **1.06** | **1.49** | **1.05** | **1.01** | **1.10** | **1.13** | **1.07** | **1.21** |
| *>84* | 1.12 | 0.95 | 1.31 | **0.55** | **0.48** | **0.64** | **1.55** | **1.35** | **1.77** | 0.74 | 0.45 | 1.20 | **1.12** | **0.96** | **1.31** | **2.13** | **1.64** | **2.77** |
| **Patient’s sex** |  |  |  |  |  |  |  |  |  |  |  |  |  |  |  |  |  |  |
| *F* | 1.00 |  |  | 1.00 |  |  | 1.00 |  |  | 1.00 |  |  | 1.00 |  |  | 1.00 |  |  |
| *M* | **0.77** | **0.73** | **0.80** | **1.19** | **1.14** | **1.24** | 1.05 | 0.99 | 1.12 | **0.80** | **0.70** | **0.91** | **0.90** | **0.86** | **0.93** | **0.89** | **0.85** | **0.93** |
| **Fee exemption** |  |  |  |  |  |  |  |  |  |  |  |  |  |  |  |  |  |  |
| *No* | 1.00 |  |  | 1.00 |  |  | 1.00 |  |  | 1.00 |  |  | 1.00 |  |  | 1.00 |  |  |
| *Yes* | **0.88** | **0.83** | **0.94** | **0.90** | **0.85** | **0.96** | 1.00 | 0.93 | 1.09 | 0.90 | 0.74 | 1.09 | **1.53** | **1.46** | **1.61** | 1.06 | 0.98 | 1.13 |
| **Physician’s sex** |  |  |  |  |  |  |  |  |  |  |  |  |  |  |  |  |  |  |
| *F* | 1.00 |  |  | 1.00 |  |  | 1.00 |  |  | 1.00 |  |  | 1.00 |  |  | 1.00 |  |  |
| *M* | **1.35** | **1.17** | **1.55** | **1.15** | **1.02** | **1.31** | 0.98 | 0.88 | 1.09 | 1.12 | 0.89 | 1.41 | 0.99 | 0.91 | 1.09 | 0.97 | 0.86 | 1.09 |
| **Physician’s age** |  |  |  |  |  |  |  |  |  |  |  |  |  |  |  |  |  |  |
| *28-44* | 1.00 |  |  | 1.00 |  |  | 1.00 |  |  | 1.00 |  |  | 1.00 |  |  | 1.00 |  |  |
| *45-68* | 1.21 | 0.93 | 1.57 | 1.19 | 0.94 | 1.51 | 1.09 | 0.87 | 1.36 | **2.09** | **1.27** | **3.45** | 0.97 | 0.81 | 1.17 | 1.10 | 0.87 | 1.39 |
| ***GPs’ practice organization*** |  |  |  |  |  |  |  |  |  |  |  |  |  |  |  |  |  |  |
| *Primary care medical group* | 1.00 |  |  | 1.00 |  |  | 1.00 |  |  | 1.00 |  |  | 1.00 |  |  | 1.00 |  |  |
| *Primary care medical network* | **1.19** | **1.02** | **1.38** | **1.23** | **1.09** | **1.40** | 1.02 | 0.92 | 1.14 | 1.23 | 0.98 | 1.55 | **1.15** | **1.04** | **1.27** | 1.06 | 0.94 | 1.21 |
| ***Year of prescription*** |  |  |  |  |  |  |  |  |  |  |  |  |  |  |  |  |  |  |
| *2012* | 1.00 |  |  | 1.00 |  |  | 1.00 |  |  | 1.00 |  |  | 1.00 |  |  | 1.00 |  |  |
| *2013* | 0.98 | 0.90 | 1.06 | **0.92** | **0.86** | **0.99** | 0.92 | 0.83 | 1.02 | 0.98 | 0.77 | 1.23 | **0.80** | **0.75** | **0.85** | 0.96 | 0.90 | 1.03 |
| *2014* | 1.00 | 0.92 | 1.09 | 0.93 | 0.87 | 1.00 | 0.93 | 0.84 | 1.03 | **0.77** | **0.62** | **0.97** | **0.82** | **0.77** | **0.87** | 0.97 | 0.90 | 1.04 |
| *2015* | 0.96 | 0.88 | 1.04 | **0.93** | **0.86** | **1.00** | 0.96 | 0.86 | 1.06 | **0.75** | **0.59** | **0.95** | **0.85** | **0.80** | **0.90** | 0.97 | 0.90 | 1.04 |
| *2016* | 0.95 | 0.87 | 1.03 | **0.82** | **0.77** | **0.89** | 0.92 | 0.83 | 1.03 | **0.69** | **0.54** | **0.88** | 0.95 | 0.90 | 1.01 | 1.02 | 0.94 | 1.10 |
| *2017* | **0.91** | **0.83** | **0.98** | **0.83** | **0.77** | **0.90** | 1.08 | 0.97 | 1.20 | **0.51** | **0.40** | **0.65** | **0.94** | **0.89** | **1.00** | 1.02 | 0.95 | 1.10 |

Appendix Table 3 **-** Multilevel analysis of the variability in inconsistence criteria or test of referral forms between GPs, primary care subdistrict and healthcare districts: random effects estimates. All referral forms made by general practitioners in the province of Reggio Emilia, Italy, between 2012 and 2017 were included.

|  | **Colonoscopy** | | **Gastroscopy** | | **Head CT** | | **Musculoskeletal CT** | | | **Head MRI** | | | **Musculoskeletal MRI** | |
| --- | --- | --- | --- | --- | --- | --- | --- | --- | --- | --- | --- | --- | --- | --- |
| *Variances of random effects** | **Variance** | **ICC %** | **Variance** | **ICC %** | **Variance** | **ICC %** | **Variance** | **ICC %** | **Variance** | | **ICC %** | **Variance** | | **ICC %** |
| Level 2 (GP) | 0.310 | 8.6 | 0.252 | 7.1 | 0.129 | 3.8 | 0.483 | 12.8 | 0.172 | | 5.0 | 0.195 | | 5.6 |
| Level 3 (primary care subdistrict) | 0.007 | 0.2 | 0.000 | 0.0 | 0.000 | 0.0 | 0.000 | 0.0 | 0.000 | | 0.0 | 0.000 | | 0.0 |
| Level 4 (healthcare district) | 0.000 | 0.0 | 0.000 | 0.0 | 0.000 | 0.0 | 0.015 | 0.0 | 0.001 | | 0.0 | 0.001 | | 0.0 |

|  | | |  |  |  |
| --- | --- | --- | --- | --- | --- |
| ** Model adjusted for 1st level covariates: Patient's Age, Sex, Exemption, year of prescription.* | | | | | |
| *ICC interclass correlation coefficient* |  |  |  |  |  |
